# Participant satisfaction across three trial arms with varying degrees of decentralisation in the RADIAL proof-of-concept trial

**DOI:** 10.1186/s13063-026-09616-4

**Published:** 2026-03-12

**Authors:** Julia Kopanz, Mira G. P. Zuidgeest, Linda Rutgrink, Cameron Keighron, Diederick E. Grobbee, Bart Lagerwaard

**Affiliations:** 1 Julius Center for Health Sciences and Primary Care, University Medical Center Utrecht, Utrecht, The Netherlands; 2 Sanofi, Amsterdam, The Netherlands; 3 International Diabetes Federation Europe, Brussels, Belgium; 4 https://trialsathome.com

**Keywords:** Participant satisfaction, Trial experience, Decentralised clinical trial, Hybrid trial, Trial innovation

## Abstract

**Background:**

As clinical trials increasingly adopt decentralised elements to potentially reduce burden and enhance participant inclusion, data on participant experience and satisfaction becomes essential to inform future decentralised trial designs. This study investigated participant satisfaction in three trial arms: a fully decentralised arm, a hybrid arm and a conventional arm in individuals with type 2 diabetes mellitus.

**Methods:**

RADIAL, a three-arm, low-intervention, phase IV proof-of-concept trial, was set up in six European countries and aimed to evaluate the scientific and operational quality as well as the feasibility of trial approaches with different levels of decentralisation. Participant satisfaction was measured at three time points: at start (week 0), during (week 12), and at the end of the trial (week 24), using digital questionnaires.

**Results:**

In all arms, 72% to 100% of participants were satisfied with overall trial participation, but satisfaction decreased over time in the hybrid arm compared to the conventional arm. Participants reported issues with trial technologies as reasons for their dissatisfaction in the hybrid arm, which was further substantiated by dissatisfaction with specific digital trial components. Satisfaction with the time commitments and data collection was also lower in the hybrid arm than in the conventional arm. Nevertheless, participants in both arms were very satisfied with the comprehensibility of the information before trial start, feeling of safety, interaction with trial staff, and timing of trial appointments, ranging from 92% to 100%. Due to the small number of seven participants in the remote arm, no meaningful conclusions on satisfaction could be drawn in this arm.

**Conclusions:**

Decentralisation seems to be acceptable to trial participants, as trial activities can be responsibly shifted from the research site to participants’ homes, e.g. decentralised, without materially impacting participant satisfaction. However, technological issues negatively affected satisfaction in the hybrid arm, highlighting the need to utilise fit-for-purpose technologies and adequately address potential digital challenges in trials. Including participant satisfaction measures in trials deepens the understanding of how participants experience different (decentralised) trial activities, which can help to tailor future trials to their needs.

**Trial registration:**

ClinicalTrials.gov NCT05780151 (2023-03-08) and CTIS 2022-500,449-26-00 (2022-03-28)

**Supplementary Information:**

The online version contains supplementary material available at 10.1186/s13063-026-09616-4.

## Background

Without people’s willingness to participate in clinical trials, research questions on efficacy and the safety of medication and other clinical interventions cannot be adequately answered [1–3]. Recruitment, however, remains a well-known challenge in clinical trials [4–7]. Trial enrolment is conditional on voluntary consent to participate after being informed about the potential risks and expectations of participation [8–10]. Once enrolled, the participant’s trial experience influences their satisfaction with the trial, which may impact their willingness to continue or to participate in another trial [11–15]. Trial participation can be perceived as burdensome by participants due to, for example, difficulties in understanding trial information, the time commitments required, interference with everyday life responsibilities, and timing and location of trial visits [1, 16–19].

In decentralised clinical trial approaches (DCTs), parts or all trial activities are shifted from the trial site to the participant’s home or close environment [20, 21], resulting in a hybrid or fully decentralised trial [21, 22]. Since DCTs place the participant at the centre of trial activities, trials can be more tailored to the daily lives of participants, possibly offering more convenient and less burdensome trial participation [23–25]. For example, participation from home may save time by reducing travel or waiting on site. Yet, remote participation may also involve less in-person contact with trial staff, requiring participants to conduct more trial tasks themselves often using digital devices [23, 25–29]. While previous studies report overall positive experiences and acceptance of decentralisation of clinical trials by participants, some also highlight clear challenges [30–36]. However, participant satisfaction is trial and context dependent, and no study to date has directly compared satisfaction with different levels of decentralisation within a single trial.

In this study, we evaluated the participant satisfaction in a fully decentralised, a hybrid and a conventional trial arm in the RADIAL proof-of-concept trial [37]. Overall satisfaction and satisfaction on individual decentralised elements were measured at three time points in the trial. The aim of this analysis was to generate empirical evidence on participant experiences with varying levels of decentralisation. Understanding patient experiences when taking part in clinical trials helps align trial activities with their needs and, when incorporated into future designs, may facilitate recruitment and retention.

## Methods

### Study design

A three-arm, low-intervention, parallel-group, open-label, 24-week, multicentre, phase IV proof-of-concept trial, namely the RADIAL (*R*emote *A*nd *D*ecentralised *I*nnovative *A*pproach to Clinical Tria*L*s) trial, was conducted in six European countries, Denmark, Germany, Italy, Poland, Spain, and the UK. The objective of the trial was to evaluate the scientific and operational quality as well as the feasibility of a remote (fully decentralised) and hybrid trial approach compared to a conventional trial approach. Several key performance indicators (KPIs) were defined to investigate this objective. A complete overview of the trial design and selection of KPIs can be found elsewhere [37]. The focus of this paper is on participant satisfaction. Individuals with type 2 diabetes mellitus (T2DM), who had basal insulin as prior medication and a 7% to 10% glycated haemoglobin (HbA1c) level, were eligible to take part in the trial.

### Ethical approvals

The RADIAL trial was performed in accordance with the principles of the Declaration of Helsinki, good scientific practice, and the EU General Data Protection Regulation. The trial was submitted in two parts through the Clinical Trials Information System (CTIS) and got authorised (2022–500,449-26-00). In the UK, the trial issued a favourable ethical opinion by both the Health Research Authority and the East of England—Essex Research Ethics Committee (IRAS ID 1006010, REC reference: 22/EE/0184). The trial was registered in the ClinicalTrials.gov database under identifier NCT05780151. All participants signed the informed consent form before trial participation.

### Set-up of the RADIAL trial

**Fig. 1:**
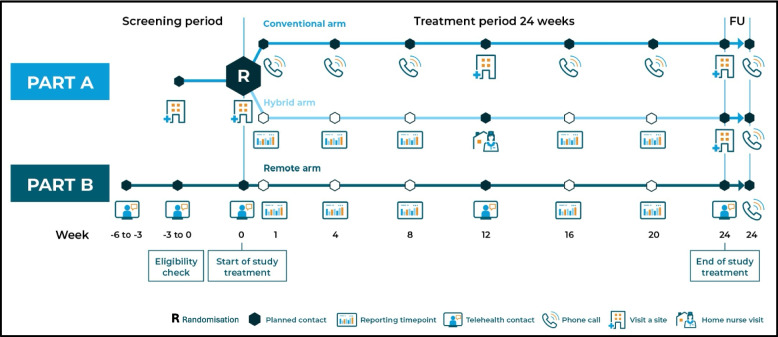
Overview of the conventional and hybrid (Part A) and remote arm (Part B) of the RADIAL trial

The RADIAL trial was designed with a total of three arms in two parts. Part A comprised a conventional arm and a hybrid arm, and Part B included a fully remote arm (Fig. 1). The three trial arms were distinguished by different decentralisation levels, i.e. the methodological intervention. In Part A, participants were recruited through conventional methods such as posters, flyers, direct outreach, and were randomised (1:1) via a randomisation and trial supply management system to either the conventional arm or the hybrid arm. In Part B, participants were recruited in a decentralised manner such as social media, search engine advertising, sites’ network, and were enrolled in the remote arm. The approach with three trial arms in two parts was chosen as it enables the testing of the fully remote design. A detailed description of the RADIAL trial design is provided by Zuidgeest et al. [37], and insights into recruitment are provided by Lagerwaard et al. [38].

While in the conventional arm visits were held on site at trial start, at week 12 and at the end of treatment, and via phone calls, participants in the hybrid arm had a combination of conventional and decentralised elements, consisting of on-site visits at trial start and at the end of treatment, a home nurse visit at week 12, and decentralised data reporting without phone calls. In contrast, participants in the remote arm never came to the site; their study visits at start, at week 12, and at the end of treatment were telemedicine visits (i.e. online visits using a videoconferencing system), and they reported their data in a decentralised manner. In all three arms, unplanned contacts in the form of trial visits were allowed when necessary, such as phone calls, telemedicine, and in exceptional cases in-person contact.

Participants in all three arms received the same treatment, i.e. clinical intervention, which was the Investigational Medicinal Product (IMP) Insulin Glargine 300 U/mL, a long-acting, marketed basal insulin analogue. A total sample size of 600 participants was targeted in the six countries, including 150 participants in the conventional arm, 150 in the hybrid arm, and 300 in the remote arm. The sample size was calculated based on a balanced ratio required to obtain meaningful insights for the exploratory analyses in this proof-of-concept trial. The trial was not powered to detect differences in all endpoints, taking into account operational, financial, and time constraints of this proof-of-concept trial in the context of the public–private research project Trials@Home.

### Data collection by participants

**Fig. 2:**
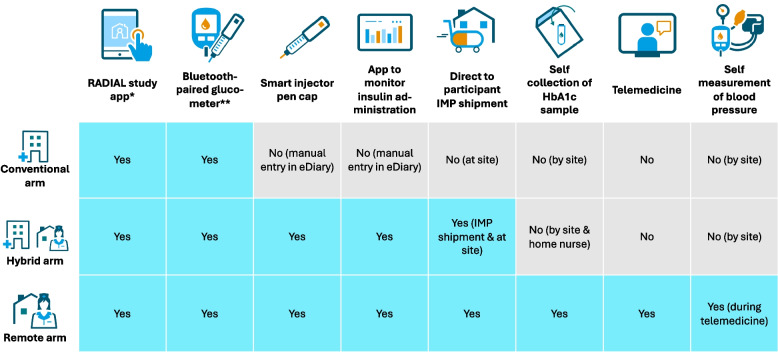
Overview of trial components and whether trial activities are performed by participants per trial arm. (IMP, Investigational Medicinal Product; HbA1c, glycated haemoglobin. *The RADIAL study app, which also acted as the eDiary, included the participant satisfaction questionnaires, electronic patient-reported outcomes (ePROs), event reporting (e.g. hypoglycaemic event), readings from bluetooth-paired glucometer, other electronic data capture and had a different configuration per trial arm. **Bluetooth connected glucometer allowed data transfer via Bluetooth to RADIAL study app.)

While the planned timing of data collection was the same in all arms (Fig. 1), some trial activities differed depending on the trial arm (Fig. 2). All participants received training, trial materials, devices, and access to the study smartphone application(s). As this was a bring-your-own-device (BYOD) study, participants in all arms installed the study app(s) on their own devices. While all participants used the study app for questionnaires, self-monitoring of plasma glucose (SMPG) via a connected glucometer, hypoglycaemia reporting, reminders, and engagement messages, additional functionalities varied by arm. In the hybrid and remote arms, the app was also used for self-reporting of medical events and changes in concomitant medication. At baseline, all participants initiated daily Insulin Gglargine 300 U/mL, the IMP, of which the daily dose was manually recorded in the app in the conventional arm and automatically captured via a smart injector pen cap in the hybrid and remote arm. In the remote arm, the app further enabled telemedicine visits, contact requests, and access to training materials. All trial materials required in the remote arm were delivered directly to participants’ homes, including the delivery of the IMP via cold-chain transport. Remote participants also completed additional self-collection activities, such as finger-prick blood samples for HbA1c and home blood pressure measurements.

### Participant satisfaction questionnaire

Participant satisfaction was collected using an adapted version of the validated TransCelerate Study Participant Feedback Questionnaire [39, 40]. A translation of the questionnaire was available in the respective languages for our study. The original phrasings of the questionnaire were used where applicable, but questions on specific decentralised trial elements were added. The Trials@Home Patient Expert Panel (PEP) and the Trials@Home External Stakeholder Panel were involved in developing these additional questions. A 5-point Likert scale ranging from strongly disagree to strongly agree was used to assess the satisfaction unless otherwise stated. Additionally, open text fields were included for some questions for participants to elaborate on their answers. Via the RADIAL study app, participants in all arms received a notification to complete the questionnaire, occurring at three time points: at the start of the trial, i.e. just after visit 2 at baseline; during the trial, i.e. just after visit 6 (12 weeks); and at the end of the trial, i.e. just after visit 9 (24 weeks) at end of treatment. Participants had a 7-day time window to complete the questionnaire, except at the end of treatment, where the questionnaire had to be filled in within 4 days after the visit. Participants were reminded twice in the 7-day period and once in the 4-day period to complete the questionnaires using push notifications on their smartphone. Only fully completed questionnaires could be submitted by participants. If participants did not complete and submit a questionnaire at one of the time points, they still received the questionnaires at the respective other time points.

For a complete overview of all questions at each time point, please see the Supplementary Materials 1, 2, and 3. In short:

- *Overall satisfaction*: Participants were asked about their overall satisfaction with trial participation.
- *Trial components*: In each of the respective trial arms, satisfaction with the use of the decentralised trial components in that arm was evaluated: the RADIAL study app, the bluetooth-paired glucometer, the smart injector pen cap, the app to monitor insulin administration, the telemedicine tool, the collection of a small blood sample, the home delivery of the study drug, and the blood pressure monitoring device (see Fig. 2). In the hybrid arm, participants were not asked about their experiences with IMP supply and HbA1c collection, because these trial activities were conducted in both the conventional (at site) and decentralised (home shipment and home nurse, respectively) way in this arm.
- *Information and trial staff contact*: Participants were asked whether the information before they joined the trial was easy to understand, and whether they had enough support from trial staff, felt comfortable to ask questions, and felt safe during the trial.
- *Time commitment and impact of (decentralised) data collection*: The acceptability of the time spent on the trial, of trial impact on daily activities, and of data collection method was assessed. Further, participants were asked whether the appointments were scheduled at a convenient time and whether the overall trial commitment required was in line with what they expected at the start.

Demographic and baseline characteristics of the participants were collected in separate questionnaires during the trial. All the questionnaires were accessible to the participants via the RADIAL study app and were fully blinded to the research site.

### Data analyses

Exploratory analyses were performed for all endpoints of the participant satisfaction KPI in this proof-of-concept trial. Data were analysed using R version 4.4.1. [41]. Frequencies and percentages were used to summarise categorical variables. Depending on the distribution, mean, standard deviation (SD), median, interquartile range, and minimum and maximum were used to present continuous variables. Only questionnaires that were submitted by participants were included in the analysis.

## Results

### Response rates and participant characteristics

A total of 108 individuals (conventional arm: *n* = 47, hybrid arm: *n* = 53, remote arm: *n* = 8) were enrolled in the RADIAL trial. High retention rates were observed in all arms (conventional arm: 91%, hybrid arm: 89%, remote arm: 75%), with four participants in the conventional arm, six in the hybrid arm, and two in the remote arm not completing the trial (Supplementary Figs. 1 and 2). Unplanned contacts occurred more often in the hybrid arm than in the conventional arm, with an average of six additional interactions between participants and sites in the hybrid arm versus two in the conventional arm. In the remote arm, an average of 10 unplanned contacts occurred.

The participant satisfaction questionnaires were sent out to active participants at each time point: at start (*n* = 107, one questionnaire was not sent out in the hybrid arm due to a technical issue), during (*n* = 99), and at end of the trial (*n* = 96). A total of 96 participants at start, 75 participants during, and 79 participants at end of the trial submitted the satisfaction questionnaire (Table 1). In Table 2, the characteristics of all trial participants and those who submitted at least one satisfaction questionnaire are listed per arm. Participants who submitted at least one satisfaction questionnaire did not differ from the overall RADIAL trial population.

**Table 1:** Satisfaction questionnaire response rates per trial arm and time point

	Response rate*		
	**Trial start**	**During the trial**	**Trial end**
**Conventional arm**	89% (42/47)	84% (36/43)	86% (37/43)
**Hybrid arm**	90% (47/52)	71% (34/48)	79% (37/47)
**Remote arm**	88% (7/8)	63% (5/8)	83% (5/6)
**Total**	90% (96/107)	76% (75/99)	82% (79/96)

*Number of questionnaires received/number of questionnaires sent out × 100%

**Table 2:** Characteristics of RADIAL participants who submitted at least one participant satisfaction questionnaire per trial arm and of all RADIAL trial participants

	**Participants who submitted at least one participant satisfaction questionnaire per arm**			All RADIAL participants (*n* = 108)*
	**Conventional arm (*n* = 46)**	**Hybrid arm (*n* = 51)**	**Remote arm (*n* = 7)**	
**Age, years** (median [min-max])	64 [33–91]	66 [40–89]	63 [53–75]	64 [33–91]
**Sex**	*n* = 46	*n* = 51	*n* = 7	*n* = 108
Male	34 (73.9%)	35 (68.6%)	4 (57.1%)	74 (68.5%)
Female	12 (26.1%)	16 (31.4%)	3 (42.9%)	33 (30.6%)
Other	-	-	-	1 (0.9%)
**Living area**	*n* = 46	*n* = 50	*n* = 7	*n* = 105
Urban	42 (91.3%)	43 (86%)	4 (57.1%)	91 (86.7%)
Rural	4 (8.7%)	7 (14%)	3 (42.9%)	14 (13.3%)
**Origin**	*n* = 44	*n* = 49	*n* = 7	*n* = 102
Asian	-	1 (2%)	-	1 (1%)
Black	-	1 (2%)	-	1 (1%)
White	41 (93.2%)	44 (89.8%)	7 (100%)	94 (92.2%)
Rather not say	3 (6.8%)	3 (6.1%)	-	6 (5.9%)
**Previous trial participation**	*n* = 46	*n* = 50	*n* = 7	*n* = 105
Yes	14 (30.4%)	22 (44%)	3 (42.9%)	40 (38.1%)
Highest level of education**	*n* = 42	*n* = 45	*n* = 6	*n* = 95
No education or primary education	-	2 (4.4%)	-	2 (2.1%)
Lower secondary education	9 (21.4%)	10 (22.2%)	2 (33.3%)	21 (22.1%)
Higher secondary education	10 (23.8%)	18 (40%)	4 (66.7%)	33 (34.7%)
Tertiary education	17 (40.5%)	9 (20%)	-	27 (28.4%)
Rather not say	6 (14.3%)	6 (13.3%)	-	12 (12.6%)
**Working life**	*n* = 42	*n* = 45	*n* = 6	*n* = 95
In paid work	12 (28.6%)	9 (20%)	1 (16.7%)	22 (23.2%)
In retirement	22 (52.4%)	29 (64.4%)	3 (50%)	56 (58.9%)
Other***	8 (19%)	6 (13.3%)	2 (33.3)	16 (16.8%)
Rather not say	-	1 (2.2%)	-	1 (1.1%)
**Access to the internet at home**	*n* = 41	*n* = 45	*n* = 6	*n* = 94
Yes	39 (95.1%)	44 (97.8%)	6 (100%)	91 (96.8%)
**Last internet use for private purposes**	*n* = 41	*n* = 45	*n* = 6	*n* = 94
Within the last 3 months	31 (75.6%)	38 (84.4%)	6 (100%)	77 (81.9%)
Between 3 months and a year ago	1 (2.4%)	2 (4.4%)	-	3 (3.2%)
More than 1 year ago	1 (2.4%)	2 (4.4%)	-	3 (3.2%)
Never used it	8 (19.5%)	3 (6.7%)	-	11 (11.7%)
**Average internet use for private purposes in the last 3 months**	*n* = 31	*n* = 38	*n* = 6	*n* = 77
Every day or almost every day	28 (90.3%)	32 (84.2%)	6 (100%)	68 (88.3%)
At least once a week (but not every day)	3 (9.7%)	6 (15.8%)	-	9 (11.7%)
**Tools used before joining RADIAL (multiple answers possible)**	*n* = 31	*n* =38	*n* = 6	*n* = 77
Digital application to record blood glucose levels	12 (38.7%)	15 (39.5%)	4 (66.7%)	31 (40.3%)
Written diary to record blood glucose levels	19 (61.3%)	18 (47.4%)	5 (83.3%)	43 (55.8%)

*A total of 108 individuals took part in RADIAL: conventional arm, *n* = 47; hybrid arm, *n* = 53; remote arm, *n* = 8. **Additional information concerning the level of education: No education or primary education (up to approximately 6 years), lower secondary education (up to approximately 9 years), higher secondary education (up to approximately 12 years), and tertiary education (bachelor’s degree or higher). ***Other occupations: Conventional arm: Self-employed (*n* = 2), unemployed (*n* = 2), not working due to illness or disability (*n* = 3), and other (*n* = 1); Hybrid arm: Self-employed (*n* = 1), unemployed (*n* = 2), not working due to illness or disability (*n* = 1), fulfilling domestic tasks and care responsibilities (*n* = 1), and other (*n* = 1); Remote arm: Not working due to illness or disability (*n* = 1) and fulfilling domestic tasks and care responsibilities (*n* = 1); All RADIAL participants: Self-employed (*n* = 3), unemployed (*n* = 4), not working due to illness or disability (*n* = 5), fulfilling domestic tasks and care responsibilities (*n* = 2), and other (*n* = 2)

### Overall participant satisfaction

**Fig. 3:**
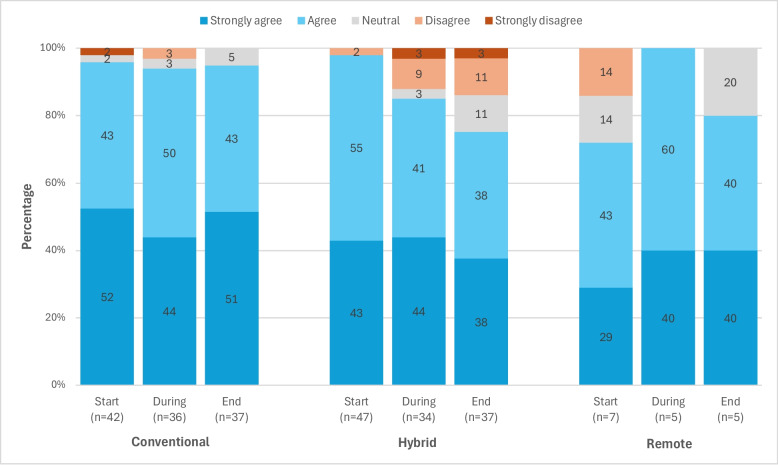
Overall satisfaction of participants in the conventional, hybrid, and remote arm at three time points: at trial start, during the trial, and at trial end (responses on a 5-point Likert scale to the question ‘Overall, I was satisfied with my trial experience’)

In all three arms, 72% to 100% of participants stated that they were very satisfied or satisfied with their overall trial participation. Over the three time points, satisfaction remained constant at 94% in the conventional arm, while it decreased from 98% to 76% in the hybrid arm. It increased in the remote arm over the three time points measured, albeit numbers in this arm were too small to draw meaningful conclusions (Fig. 3). However, when asked in a separate question at trial end whether they were interested in participating in other decentralised trials, all participants in the remote arm expressed interest in participating in such trials.

As reasons for their satisfaction, very satisfied and satisfied participants in the conventional and hybrid arms at all three time points primarily mentioned the following aspects: general contentment, improvement of diabetes control, improvement and monitoring of health, explanation of information, service quality, and contact with trial staff. In both arms, some satisfied participants also stated technical problems, with these being mentioned more often towards the end of the trial. The organisation of trial activities and appointments was also mentioned as a reason for their satisfaction by very few participants in both arms at trial start. During the trial, very few participants in the hybrid arm named altruistic motivations such as helping others. Participants who were very dissatisfied, dissatisfied, or neutral in the hybrid arm mainly mentioned technical issues, such as problems with the app or devices. In the conventional arm, only very few participants were very dissatisfied, dissatisfied, or neutral. They reported technical problems, but also other reasons, such as no improvement in health. Due to the small number of observations in the remote arm, no main themes could be identified, but the responses were similar to those reported by participants in the other two arms.

### Satisfaction with trial components

**Fig. 4:**
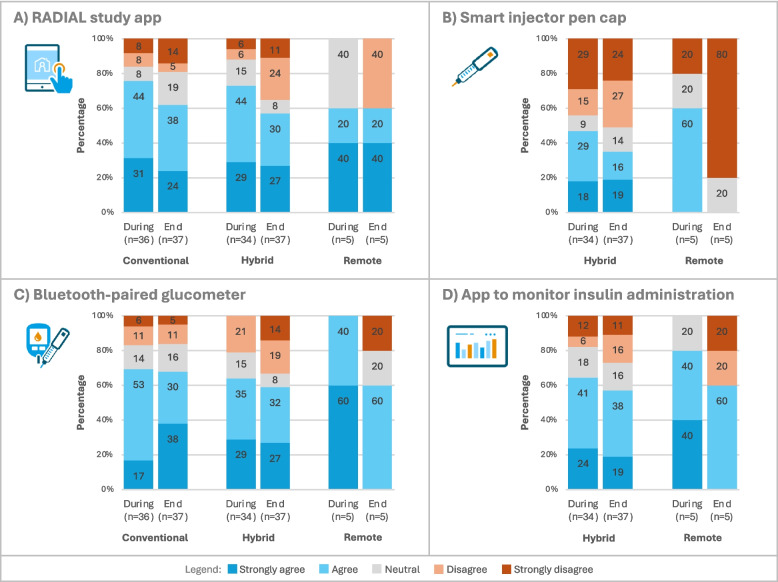
Satisfaction with the use of the RADIAL study app (**A**), the smart injector pen cap (**B**), the bluetooth-paired glucometer (**C**), and the app to monitor insulin administration (**D**) during and at the end of the trial for the three trial arms (responses on a 5-point Likert scale to the question ‘I was satisfied with using the study component’)

In Fig. 4, the satisfaction levels for the various trial components are shown. Around three quarters of participants in the conventional and hybrid arms were very satisfied or satisfied with the RADIAL study app during the trial. This decreased to 62% in the conventional arm and 57% in the hybrid arm at trial end. Among participants in the remote arm, although small in number, satisfaction with the RADIAL study app remained constant at 60% but declined for all other trial components by the trial end. Regarding the bluetooth-paired glucometer, satisfaction during the trial was higher in the conventional arm when compared to the hybrid arm (70% vs. 64%) and declined to 68% and 59%, respectively, at trial end. In the hybrid arm, satisfaction with the insulin monitoring app was 65% and with the smart injector pen cap 47% during the trial, with both decreasing to 57% and 35%, respectively, by the trial end.

Reasons for dissatisfaction with the trial components were mainly related to app and device malfunctions as well as connection issues with the glucometer. For the study app, a few participants noted difficulty with the use of the app, including problems with data entry and data presentation of the trial components, e.g. displaying the most recent blood glucose levels at the bottom of the page, different units displayed on the glucometer and the application. For the smart injector pen cap at both time points, participants mainly mentioned device malfunctions and connection problems, which caused some participants to revert to entering their data manually in the study app.

Participants in the remote arm, albeit few in numbers, were very satisfied or satisfied with the following decentralised trial components used in this arm which are as follows: the delivery of the study drug at home (40% strongly agree, 60% agree, during trial), the telemedicine tool for interaction moments (100% agree, at trial end), and the blood pressure monitoring device (100% strongly agree, at trial end). A lower satisfaction was reported with the self-collection of a small blood sample by finger prick and its shipment to the laboratory by participants in the remote arm (40% strongly agree, 20% agree, 40% neutral, during trial).

### Satisfaction with information and trial staff contact

**Fig. 5:**
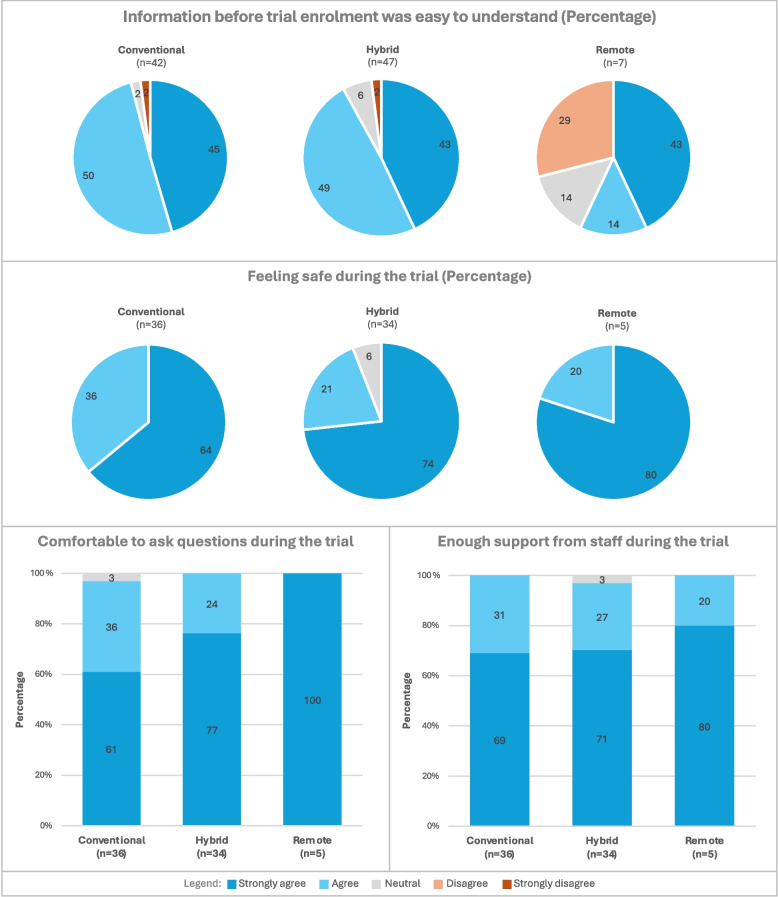
Satisfaction with the information before trial enrolment, feeling safe, and satisfaction with trial staff contact during the trial in the three trial arms

Almost all participants in all arms were very satisfied or satisfied with the level of support experienced from trial staff, felt comfortable with asking questions and felt safe during the trial (Fig. 5). The majority of participants in the conventional and hybrid arms found the information provided prior to trial start (e.g. visits and procedures, time commitment, who to contact with questions) easy to understand (95% and 92%). In the remote arm, although small in number, 57% of participants thought the information before trial enrolment was easy to understand.

### Impact of time commitment and of (decentralised) data collection on satisfaction

**Fig. 6:**
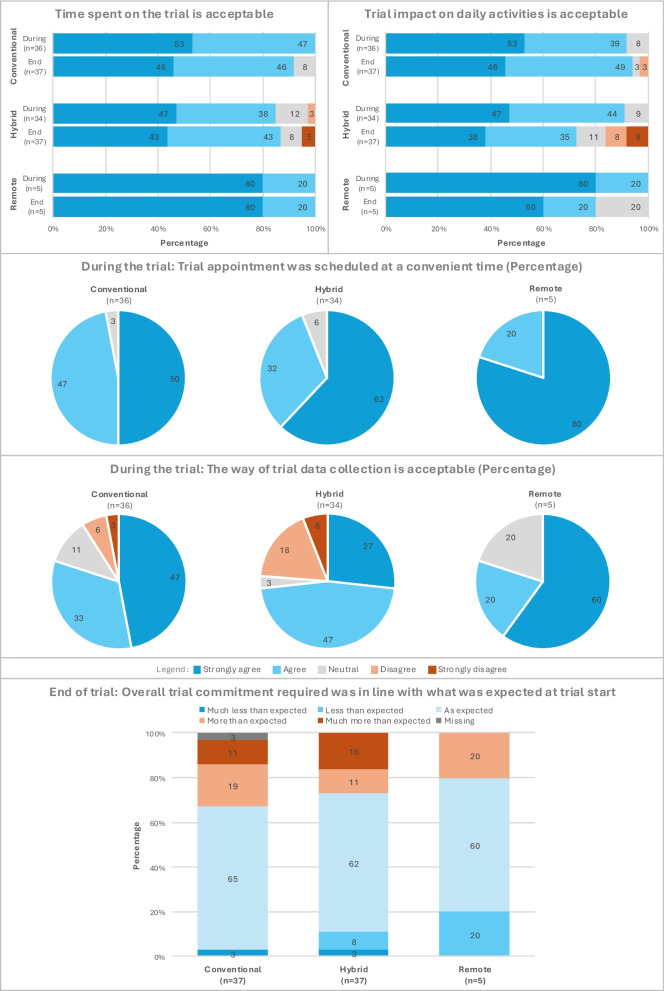
Satisfaction with time commitment and impact of (decentralised) data collection during and at the end of the trial for the three trial arms

In all arms, almost all participants were very satisfied or satisfied with the timing of trial appointments (Fig. 6). A total of 73% to 100% of participants were very satisfied or satisfied with the time spent on the trial, the impact of the trial on their daily activities, and the data collection mode (e.g. questionnaires, eDiary, glucometer, technology). Dissatisfaction was highest in the hybrid arm with regard to the impact on everyday activities (16%) and the method of data collection (24%). Most participants in all arms felt that the overall commitment required for the trial was in line with their expectations at baseline (60%–65%). For all aspects except overall required trial commitment, participants in the conventional arm reported slightly higher satisfaction than those in the hybrid arm.

## Discussion

This study aimed to evaluate the participant satisfaction in a fully decentralised, a hybrid and a conventional trial arm for participants who took part in the RADIAL trial. Overall satisfaction with trial participation was high and remained stable in the conventional arm but declined from start to end in the hybrid arm. In the remote arm, the number of participants was too low to draw meaningful conclusions. The decline in satisfaction in the hybrid arm seems to mainly originate from technology malfunctions and connection problems, as shown from the participants’ explanation for their overall satisfaction score and the dissatisfaction with specific trial components. Yet, although technical problems occurred and negatively affected satisfaction, other critical aspects of participant satisfaction, such as the comprehensibility of trial information, feeling of safety, interaction with trial staff, and timing of trial appointments, obtained equal levels of satisfaction in both the conventional and hybrid arms and were thus neither increased nor decreased by decentralisation. Decentralisation therefore seemed to be acceptable to trial participants, but a higher satisfaction, which may have been expected given the proposed advantages of DCTs for participants, was not observed.

Similarly to our study, other DCTs reported technology-related issues [30, 34, 35, 42]. Such technology issues may arise from different causes, such as limited digital literacy, or problems with the software or hardware itself [43–47]. In RADIAL, the Trials@Home PEP members were actively involved in evaluating and selecting the technology used and its implementation in the study. All the technology deployed was validated and verified, but some of the devices frequently malfunctioned during the trial. Further, issues with the interoperability and compatibility of the devices with the participants’ smartphones were encountered in this BYOD trial [48]. This suggests that the reasons for the discontent, as reflected in participants’ answers, were likely due to true software and hardware issues, and not due to participants’ inability to operate the device. To prevent device issues in future trials, it is essential not only to use validated and verified digital tools [49–52] but also to ensure their interoperability and compatibility with the wide array of combinations of hardware and operating systems encountered in the real world [48]. Further, a risk management plan is recommended, including anticipated technical issues and mitigation strategies [49–52]. Besides that, participants need to know who to contact in case of technical issues, as stressed in guidance documents [49, 50, 52], and technology support, for example in the form of a centralised dedicated support system, is advised [48]. The learnings concerning technological challenges in the RADIAL trial are provided in detail by Hanke et al. [48], and we recommend considering those in the planning of future trials.

Participants in the hybrid arm also expressed lower satisfaction with time demands, daily life impact, and data collection compared to participants in the conventional arm. This might be due to disappointments because expectations were not met or extra time spent, for example, on troubleshooting, reaching out for support for technology issues or the shift to manual data entry for some participants. However, the use of digital devices may also decrease burden on participants in DCTs [27]. Participants in conventional trials often complain about travel, waiting time on site, or timing of trial visits [16, 19], whereas the experiences of participants in DCTs highlight the convenience of participating from their home [32, 34]. Hence, it can be assumed that the benefit and comfort of not having to travel to the research site may outweigh the burden arising from technology issues for some participants. Our results also showed that the percentage of participants in the hybrid and remote arm who were very satisfied with the timing of their scheduled trial appointments was higher than in the conventional arm, which was likely due to the flexibility of home visits and video calls. Nevertheless, satisfaction with the timing of trial appointments was almost equal in the conventional and hybrid arm when very satisfied and satisfied participants were combined.

An equally positive evaluation was also obtained for the interaction with trial staff (i.e. comfortable to ask questions and having enough support from trial staff), the comprehensibility of the information before trial enrolment, and the feeling of safety in the hybrid and the conventional arm. In the remote arm, although numbers were low, high satisfaction with interaction with trial staff and feeling of safety was also observed. A smaller percentage of participants found the information provided prior to trial start easy to understand in the remote arm, but reasons for the responses were not asked. Our findings suggest that full or partial remote contact is just as acceptable to participants as exclusively in-person contact on site. Concerns regarding both the mode and frequency of contact with trial staff being challenging for participants in DCTs [26, 29] are hence not supported by our findings. It is to be noted that this may partly be explained by the fact that RADIAL allowed unplanned contacts, which occurred more often in the hybrid arm compared to the conventional arm. Most of the unplanned contacts in the hybrid arm were initiated by the site and were triggered by device malfunctions, additional training needs, participants not submitting data at the expected time points, and disease management (e.g. insulin dose adjustment, discussion of hypoglycaemic events) [53]. Adding this to the number of planned contacts (4 and 9 in the hybrid and conventional arm, respectively), this results in an almost similar amount of interaction times per participant in both arms. This room for additional interaction with trial staff when needed may have contributed to the satisfaction with trial staff interactions in the hybrid arm. In other DCTs, positive experiences with trial staff interactions were reported too [30, 33, 35], although some participants wished to increase contact moments [30, 33].

### Strengths and limitations

A strength of this study is its design, which allows for the investigation of satisfaction with trial participation in arms with different levels of decentralisation within the same population and the same clinical intervention, enabling a direct comparison of satisfaction with decentralised elements between the conventional and the hybrid arm. A major limitation is the small number of participants in the remote arm, not allowing clear conclusions on this group. In short, the under-recruitment in RADIAL can be explained by a general lack of interest in the trial among patients and limited perceived benefits for patients by sites, given the methodological nature of the proof-of-concept trial, but also by other factors, including that recruitment in Part B took place at a single site per country and that some recruitment strategies, such as direct patient outreach, were more successful than others, such as online recruitment [38].

The satisfaction questionnaire response rate between 71% and 90% in the conventional and hybrid trial arms at different time points indicates that the adapted version of the TransCelerate Study Participant Feedback Questionnaire [39, 40] was well accepted by participants. A small number of missing data were reported in our study as some participants discontinued their participation. Since these participants were not explicitly asked about their satisfaction at that point in time, there is no information available regarding their satisfaction or its potential impact on their decision to leave. This research focuses on the experiences of individuals with T2DM. Our study findings are expected to be generalisable to the general diabetes population, which is comparable to our population in terms of age and sex. While our findings on satisfaction with decentralised elements may also be generalisable to other chronic diseases, the results might not apply to patients living with other indications, especially when familiarity with disease self-management and digital technologies is less common.

## Conclusions

The advantages often promised with decentralisation of clinical trials could lead to a higher satisfaction among participants taking part in hybrid and remote trial designs compared to conventional ones. However, this was not the case in the current study evaluating trial participation satisfaction in trial arms with varying degrees of decentralisation. A lower satisfaction was observed in the hybrid arm compared to the conventional arm, mainly due to technical issues with devices used in the hybrid arm. Despite this, decentralisation of trial conduct seemed to be acceptable to trial participants; even though technologies decreased satisfaction when they did not function as intended, participants in the hybrid and conventional arm were equally highly satisfied with the comprehensibility of trial information, the feeling of safety, their contact with trial staff, and the timing of trial appointments, suggesting that trial activities can be responsibly moved from sites to participants’ homes. It is, however, recommended that the technology is fit-for-purpose, and that measures are in place to address potential technological challenges. To continuously enhance and take the experiences of participants in DCTs forward, the implementation of satisfaction measures is encouraged in future trials. Attention to patients’ needs and experiences in DCTs will contribute to better recruitment, retention, and diversity among trial participants.

## Supplementary Information

Supplementary Material 1: Satisfaction questionnaire at the beginning of the trial. Supplementary Material 2: Satisfaction questionnaire during the trial. Supplementary Material 3: Satisfaction questionnaire at the end of the trial. Supplementary Material 4: Supplementary Figure 1: Participant flow for Part A; Supplementary Figure 2: Participant flow for Part B.

## Data Availability

The data collected and analysed during the current study are available from the corresponding author upon reasonable request.

## Abbreviations

BYOD: Bring your own device
CTIS: Clinical Trials Information System
DCTs: Decentralised clinical trial approaches
ePROs: Electronic patient-reported outcomes
HbA1c: Glycated haemoglobin
IMP: Investigational Medicinal Product
KPIs: Key performance indicators
RADIAL: *R*emote *A*nd *D*ecentralised *I*nnovative *A*pproach to Clinical Tria*L*s
SD: Standard deviation
SMPG: Self-monitoring of plasma glucose
T2DM: Type 2 diabetes mellitus
