# Supplementary material for: Participant satisfaction across three trial arms with varying degrees of decentralisation in the RADIAL proof-of-concept trial
Source: Trials. 2026 Mar 12;27:311. doi: 10.1186/s13063-026-09616-4 (PMC13094246; doi:10.1186/s13063-026-09616-4)
Supplement: Supplementary file 1 — Supplementary Material 1: Satisfaction questionnaire at the beginning of the trial. Supplementary Material 2: Satisfaction questionnaire during the trial. Supplementary Material 3: Satisfaction questionnaire at the end of the trial. Supplementary Material 4: Supplementary Figure 1: Participant flow for Part A; Supplementary Figure 2: Participant flow for Part B. [file 13063_2026_9616_MOESM1_ESM.docx]

**Supplementary Material**

**Participant satisfaction across three trial arms with varying degrees of decentralisation in the RADIAL proof-of-concept trial**

Julia Kopanz^1^, Mira G.P. Zuidgeest^1^, Linda Rutgrink^2^, Cameron Keighron^3^, Diederick E. Grobbee^1^, Bart Lagerwaard^1^, on behalf of the Trials@Home consortium^4^

1 Julius Center for Health Sciences and Primary Care, University Medical Center Utrecht, Utrecht, the Netherlands

2 Sanofi, Amsterdam, the Netherlands

3 International Diabetes Federation Europe, Brussels, Belgium

4 trialsathome.com

**Content**

**Supplementary Material 1: Satisfaction questionnaire at the beginning of the trial**

**Supplementary Material 2: Satisfaction questionnaire during the trial**

**Supplementary Material 3: Satisfaction questionnaire at the end of the trial**

**Supplementary Material 4: Supplementary Figure 1: Participant flow for Part A; Supplementary Figure 2: Participant flow for Part B**

**Supplementary Material 1: Satisfaction questionnaire at the beginning of the trial**

# Section A: Your experience in the beginning of the study

## Expected after Visit 2 (baseline)

This questionnaire should not be used to provide immediate feedback to the study team. If you need to address any immediate concerns or issues, please reach out to your investigator directly.

|  | **Conventional** | **Hybrid** | **Remote** | Answer pattern |
| --- | --- | --- | --- | --- |
| 1 | Overall, I am satisfied with my trial experience so far  What is the most important reason(s) for your answer? | | | LS-5* |
| 2 | I understand the treatment process in this trial (for example: when and how to take or use a treatment) | | | LS-5 |
| 3 | The information given to me before I joined the trial was everything I wanted to know about the trial (for example: visits and procedures, time commitment, who to contact with questions)   - [If participant replied "Strongly disagree" or "Disagree" prompt: Please provide more details to explain the reasons for your answer] | | | LS-5 |
| 4 | The information given to me before I joined the trial was easy to understand (for example: visits and procedures, time commitment, who to contact with questions) | | | LS-5 |
| 5 | I felt comfortable that I could ask any questions before I joined the trial | | | LS-5 |
| 6 | I understand what the expected commitment for this trial will be | | | LS-5 |
| 7 | I was adequately informed on how to use the following study components: | | |  |
| a | The RADIAL study app to report my data (for example, questionnaires and hypoglycemic events) | | | LS-5 |
| b | - | - | The telemedicine tool for interaction moments (Virtrial) | LS-5 |
| c | - | The collection of the dose and timing of study drug using a smart injector pen cap | The collection of the dose and timing data using a smart injector pen cap | LS-5 |
| d | - | The visualization of the dose and timing data in a mobile app (MEMS app) | The visualization of the dose and timing data in a mobile app (MEMS app) | LS-5 |
| e | The bluetooth-paired glucometer for collection of fasting glucose measurements | | | LS-5 |
| f | - | - | The collection of a small blood sample for shipment via the mail | LS-5 |
| g |  |  | The delivery of the study drug at your house |  |
|  | [If participant replied "Strongly disagree" or "Disagree" prompt: Please provide more details to explain the reasons for your answer] | | |  |

**LS-5: Likert Scale 5 point, Strongly Disagree – Strongly Agree*

**Supplementary Material 2: Satisfaction questionnaire during the trial**

# Section B: Your experience during the trial

## Expected at Visit6

|  | **Conventional** | **Hybrid** | **Remote** | Answer pattern |
| --- | --- | --- | --- | --- |
| 1 | Overall, I am satisfied with my trial experience so far  What is the most important reason(s) for your answer? | | | LS-5* |
| 2 | My scheduled trial appointments (for example: visits, calls, telehealth contact) have been well organized | | | LS-5 |
| 3 | My scheduled trial appointments (for example: visits, calls, telehealth contact) are scheduled at a convenient time for me | | | LS-5 |
| 4 | The staff treats me with respect | | | LS-5 |
| 5 | I feel comfortable that I can ask questions during the trial | | | LS-5 |
| 6 | I am satisfied with the answers I have received to my questions during the trial | | | LS-5 |
| 7 | I feel I have enough support from the trial staff   - [If participant replied "Strongly disagree" or "Disagree" prompt: Please provide more details to explain the reasons for your answer] | | | LS-5 |
| 8 | I feel safe during this trial   - [If participant replied "Strongly disagree" or "Disagree" prompt: Please provide more details to explain the reasons for your answer] | | | LS-5 |
| 9 | The time spent on the study is acceptable to me | | | LS-5 |
| 10 | The impact the trial has on my daily activities is acceptable to me (for example: household chores, work, commitments, eating) | | | LS-5 |
| 11 | The way in which trial data is being collected is acceptable to me (for example: online, questionnaires, e-diary, glucometer, technology, in person) | | | LS-5 |
| 12 | I am being kept informed of the results of my medical tests done during the trial, including during screening (for example: blood tests) | | | LS-5 |
| 13 | I am satisfied with using the following study component: | | |  |
| a | The RADIAL study app to report my data (for example, questionnaires and hypoglycemic events) | | | LS-5 |
| b | - | - | A telemedicine tool for interaction moments (Virtrial) | LS-5 |
| c | - | The collection of the dose and timing of study drug using a smart injector pen cap | The collection of the dose and timing data using a smart injector pen cap | LS-5 |
| d | - | The visualization of the dose and timing data in a mobile app (MEMS app) | The visualization of the dose and timing data in a mobile app (MEMS app) | LS-5 |
| e | The bluetooth paired glucometer for collection of fasting glucose measurements | | | LS-5 |
| f | - | - | The collection of a small blood sample for shipment via the mail | LS-5 |
| g |  |  | The delivery of the study drug at your house |  |
|  | - [If participant replied "Strongly disagree" or "Disagree" prompt: Please provide more details to explain the reasons for your answer] | | |  |
| 14 | I find the following study component easy to use: | | |  |
| a | The RADIAL study app to report my data (for example, questionnaires and hypoglycemic events) | | | LS-5 |
| b | - | - | A telemedicine tool for interaction moments (Virtrial) | LS-5 |
| c | - | The collection of the dose and timing of study drug using a smart injector pen cap | The collection of the dose and timing data using a smart injector pen cap | LS-5 |
| d | - | The visualization of the dose and timing data in a mobile app (MEMS app) | The visualization of the dose and timing data in a mobile app (MEMS app) | LS-5 |
| e | The bluetooth-paired glucometer for collection of fasting glucose measurements | | | LS-5 |
| f | - | - | The collection of a small blood sample for shipment via the mail | LS-5 |
| g |  |  | The delivery of the study drug at your house |  |

**LS-5: Likert Scale 5 point, Strongly Disagree – Strongly Agree*

**Supplementary Material 3: Satisfaction questionnaire at the end of the trial**

# Section C: Your experience at the end of the trial

## Expected at Visit9 (End of Trial)

|  | **Conventional** | **Hybrid** | **Remote** | Answer pattern |
| --- | --- | --- | --- | --- |
| 1 | Overall, I was satisfied with my trial experience  What is the most important reason(s) for your answer? | | | LS-5* |
| 2 | Overall, I was satisfied with the information I received about future support after the trial (for example: future treatment, follow-up contact details) | | | LS-5 |
| 3 | The overall commitment required for the trial was in line with what I expected when I started the trial | | | Likert scale,  5 points,  Much less than expected – Much more than expected |
| 4 | The time spent on the study was acceptable to me (for example: scheduled trial appointments, questionnaires, forms) | | | LS-5 |
| 5 | The impact the trial had on my daily activities was acceptable to me (for example: household chores, work, commitments, eating) | | | LS-5 |
| 6 | I have been kept informed of the results of my medical tests done during the trial, including during screening (for example: blood tests) | | | LS-5 |
| 7 | I feel I had enough support from the trial staff  - If you replied "Strongly disagree" or "Disagree", please provide more details to explain the reasons for your answer | | | LS-5 |
| 8 | I felt safe during this trial  - If you replied "Strongly disagree" or "Disagree", please provide more details to explain the reasons for your answer | | | LS-5 |
| 9 | I was satisfied with using the following study component: | | |  |
| a | The RADIAL study app to report my data (for example, questionnaires and hypoglycemic events) | | | LS-5 |
| b | - | - | A telemedicine tool for interaction moments (the Virtrial app) | LS-5 |
| c | - | The collection of the dose and timing of study drug using a smart injector pen cap | The collection of the dose and timing data using a smart injector pen cap | LS-5 |
| d | - | The visualization of the dose and timing data in a mobile app (MEMS app) | The visualization of the dose and timing data in a mobile app (MEMS app) | LS-5 |
| e | The bluetooth-paired glucometer for collection of fasting glucose measurements | | | LS-5 |
| f | - | - | The blood pressure monitoring device | LS-5 |
|  | - [If participant replied "Strongly disagree" or "Disagree" prompt: Please provide more details to explain the reasons for your answer] | | |  |
| 10 | I found the following study component was easy to use: | | |  |
| a | The RADIAL study app to report my data (for example, questionnaires and hypoglycemic events) | | | LS-5 |
| b | - | - | A telemedicine tool for interaction moments (the Virtrial app) | LS-5 |
| c | - | The collection of the dose and timing data using a smart injector pen cap | The collection of the dose and timing data using a smart injector pen cap | LS-5 |
| d | - | The visualization of the dose and timing data in a mobile app (MEMS app) | The visualization of the dose and timing data in a mobile app (MEMS app) | LS-5 |
| e | The bluetooth paired glucometer for collection of fasting glucose measurements | | | LS-5 |
| f | - | - | The blood pressure monitoring device | LS-5 |
| 11 | I was informed of any future opportunities to access the overall trial results if I wanted to | | | Binary – No/Yes |
| 12 | - | - | If you could, would you take part in other online trials? | LS-5 |

**LS-5: Likert Scale 5 point, Strongly Disagree – Strongly Agree*

**Supplementary Material 4**


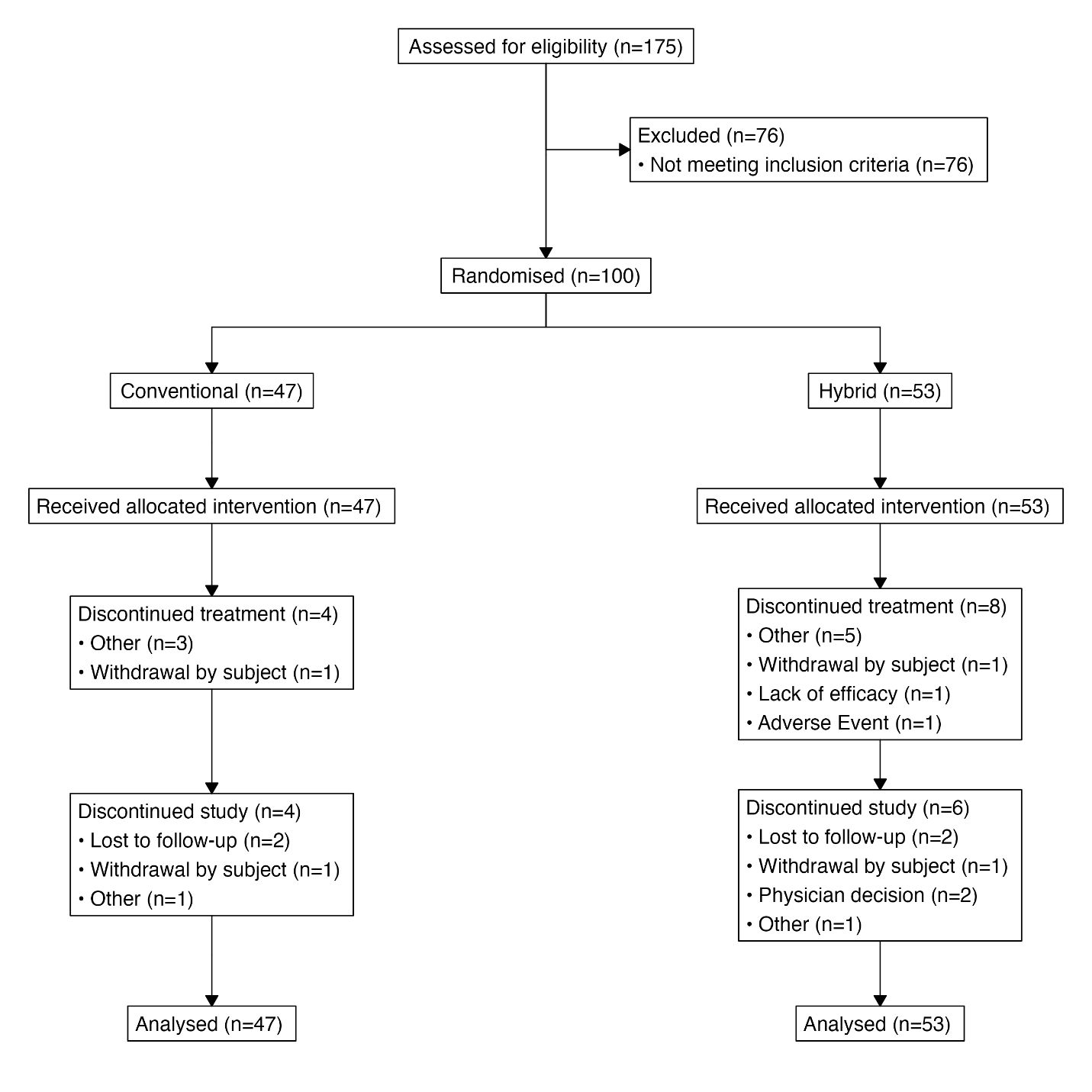


**Supplementary Figure 1** Participant flow for Part A


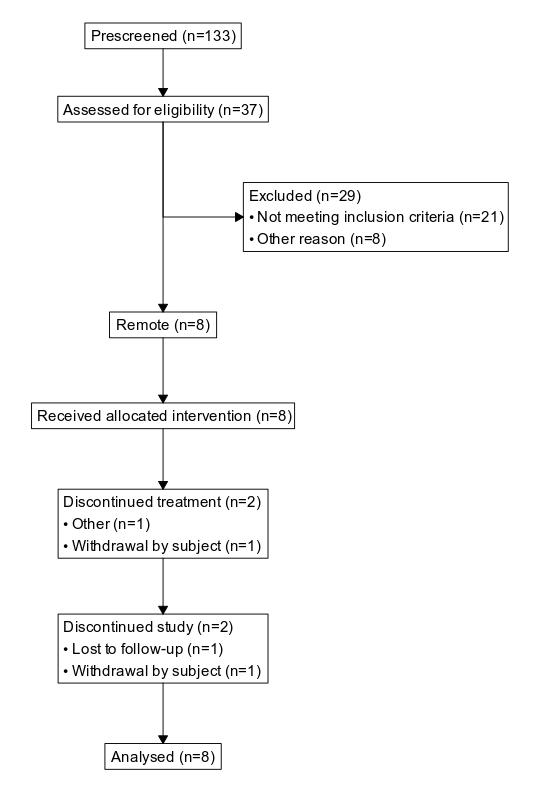


**Supplementary Figure 2** Participant flow for Part B
